# The use of inappropriate anal douching tool associates with increased HIV infection among men who have sex with men: a cross-sectional study in Shenyang, China

**DOI:** 10.1186/s12889-021-10276-z

**Published:** 2021-01-28

**Authors:** Zhen Xing Chu, Guangquan Shen, Qinghai Hu, Hongyi Wang, Jing Zhang, Willa Dong, Yongjun Jiang, Wenqing Geng, Hong Shang, Junjie Xu

**Affiliations:** 1 NHC Key Laboratory of AIDS Immunology (China Medical University), National Clinical Research Center for Laboratory Medicine, The First Affiliated Hospital of China Medical University, No. 155, Nanjingbei Street, Heping District, Shenyang, 110001 Liaoning Province China; 2 Key Laboratory of AIDS Immunology of Liaoning Province, The First Affiliated Hospital of China Medical University, Shenyang, 110001 China; 3 Key Laboratory of AIDS Immunology, Chinese Academy of Medical Sciences, Shenyang, 110001 China; 4 Collaborative Innovation Center for Diagnosis and Treatment of Infectious Diseases, 79 Qingchun Street, Hangzhou, 310003 China; 5 The Southern Medical University Institute for Global Health and Sexually Transmitted Diseases, Guangzhou, China; 6 SESH Global, Guangzhou, China; 7 University of North Carolina Project-China, Guangzhou, China; 8 Department of Epidemiology, University of North Carolina at Chapel Hill, Chapel Hill, NC USA

**Keywords:** Men who have sex with men (MSM), Rectal douching (RD), Serosorting, Sexual roles

## Abstract

**Background:**

Rectal douching (RD) is widely practiced by men who have sex with men (MSM), and is associated with increased risk of HIV infection. However, the mechanism of how RD increases the risk of HIV infection is not well understood, and there is limited data on RD behavior in MSM practicing anal sex in China. We examine the purpose of RD, its timing in relation to anal sex, the types of RD products used, and risky sexual behaviors among MSM reporting anal sex.

**Methods:**

Between August 2017 and December 2018, a cross-sectional study was conducted among adult MSM in Shenyang, China. Data were collected on demographics, sexual behaviors, and RD for the most recent sexual intercourse by means of interviewer-administered face-to-face questionnaires. Blood samples were collected to test for antibodies to HIV and syphilis. Multivariable logistic regression models were used to assess the risk factors associated with HIV infection.

**Results:**

A total of 515 eligible MSM participated in this survey (median age: 31 years). During the most recent anal intercourse, 28.3% (146/515) had condomless receptive anal intercourse (CRAI), 21.4% (110/515) practiced serosorting, and more than half (61.6%, 317/515) reported RD before or after anal sex. Of those practicing RD, 96.8% (307/317) conducted RD before sex, while 62.5% (198/317) conducted RD after sex. The douching devices used were primarily shower hoses (85.3%, 262/307), and relatively few MSM used commercial RD products (8.1%, 25/307) before sex. The prevalence of HIV-1 and syphilis was 11.7% and 13.2%, respectively. HIV infection was positively associated with RD, practicing RD before sex, the interaction between RD and CRAI using a shower hose for RD and other risk factors, practicing RD after sex, CRAI, using nitrite inhalants, main sexual role with males as bottom and syphilis infection.

**Conclusions:**

RD is popular among Chinese MSM. Improper noncommercial RD tools use (such as shower hose), the interaction effect between RD and CRAI associated with HIV infection. Public health workers and the MSM community should publicize scientific knowledge and prevention approaches relating to RD and HIV transmission to MSM. We recommend that further studies should be conducted to understand the detailed mechanism between RD and increased HIV prevalence.

**Supplementary Information:**

The online version contains supplementary material available at 10.1186/s12889-021-10276-z.

## Background

HIV/AIDS is a serious global health concern, with 38.0 million people estimated to be living with HIV in 2019 around the world [1]. By the end of 2018, China had around 1.25 million people living with HIV [2]. The risk of getting HIV is 26 times higher among MSM compared to other populations. The proportion of newly diagnosed HIV infections contracted through male same-sex intercourse rapidly increased from 2.5% in 2006 to 25.5% in 2017 in China [3]. Moreover, China has an estimated MSM population of 18 million, with the average prevalence of HIV 4.9% [4, 5]. HIV is still a fatal disease, and HIV can cause lung cancer and other deadly tumors, reduce the life expectancy of infected MSM [4, 6–8]. Meanwhile, contracting syphilis increases HIV infections for MSM. A study conducted from 2000 to 2013 in 27 high-income countries found the syphilis infection rate among MSM grew from 26.8 to 55.0% [9]. The syphilis infection rate among MSM in China was estimated to be 11.8% in 2013, based on a study in 61 Chinese cities [5].

The World Health Organization (WHO) has proposed treatment as prevention (TasP), post-exposure prophylaxis (PEP), pre-exposure prophylaxis (PrEP) and behavior intervention strategies, etc. to control the HIV epidemic. However, the HIV epidemic among MSM populations in most countries around the world has not been effectively controlled [10]. Understanding the sexual behaviors and preferences of this key population group is essential for developing effective prevention strategies [11]. In recent years, the rectal douching (RD) behavior of MSM has received widespread attention. RD involves injecting various kinds of liquid into the rectum using tools to facilitate defecation and cleansing of the rectum. This behavior is common among MSM globally, both before and after anal sex. In the previous studies, around 66 and 63% of MSM in the USA and Kenya, respectively, were found to have conducted RD. [12, 13] The percentages of MSM employing RD hover around 53.4–54.0% in the UK, Brazil, and France [11, 14, 15]. In Peru, the Netherlands, and China (Beijing City), the prevalence of RD among MSM is 18.2–27.0%, 13.6–46.0, and 59.0%, respectively. Around a quarter of MSM in the USA did not know how to douche the rectum correctly [16], and more than 94% of MSM in Brazil had not received any professional instruction [14]. These reports indicate that RD is a widespread practice among the key populations vulnerable to HIV infection. If ignored, this behavior may result in increased transmission of HIV.

Despite the reported association between RD and increased HIV infection, RD’s mechanism contributes to HIV infection remains unclear [17]. RD timing and the types of RD tools used are two factors that may affect the HIV infection risk of MSM employing this practice. Percentages of MSM using RD is most frequently practiced before anal sex 87–97%, with only 13–48% conducting RD post-anal intercourse [18]. Considering this disparity between pre- and post-sex RD practice, it is essential to distinguish between the timings of RD in order to evaluate their impact on HIV infection. The main solutions used for RD are tap water and homemade solutions (e.g., water and soap) [14, 19]. For the difference in fluid osmotic pressure inside and outside the rectal epithelial cells, the latter are in a fully filled state following RD, and the risk of rupturing them is increased during anal sex. Water-based enema solutions are typically hypotonic and exert a lower osmotic pressure compared to the contents of the colon. As a result, excess water may be absorbed by epithelial cells, leading to water toxicity and cell lysis [20]. Currently, it is still unknown whether the timing of RD correlates with the rates of HIV infection among MSM. Also, RD is a complex behavior that may involve various douching tools, including a shower hose, a plastic pump, and plastic bottles [14], which can be either commercial products or noncommercial/homemade products (also called inappropriate douching tools) [19]. Even RD and other risky sexual behavior such as CARI has a positive relationship with the HIV infection [17, 21]. Whether these factors can interact with each other is still not clear while knowing this is vital for designing corresponding interventions for MSM. Until now, only scant attention has been paid to the association between the use of different kinds of douching tools and HIV infection, and there is a general lack of data on RD in China.

We, therefore, conducted a cross-sectional study to investigate the relationship between RD and risk behavior with HIV infection and examine whether there exists an interaction effect between RD and risk behavior on HIV infection among MSM. So we could promote better, safer-sex education and design useful HIV prevention guidelines in the MSM community.

## Methods

### Study design, duration and population

Between September 2017 and December 2018, we conducted a descriptive cross-sectional study. MSM respondents were deemed eligible to participate in the study if they were: (i) over 18 years old, (ii) MSM who reported having had anal or oral sex within the previous 6 months, (iii) MSM who agreed to be tested for HIV and disclose information about their most recent experience of sexual intercourse, and (iv) were willing and able to sign a written informed consent document. Subjects who had previously tested HIV-positive were excluded from the study.

### Study setting

This study was completed in Shenyang, a politico-economic-cultural center in northeastern China and the provincial capital of Liaoning Province. Shenyang’s gross domestic product (GDP) ranked 34th of the 100 largest cities in China in 2018. Shenyang has a population of over 8.1 million, with an estimated 140,000 MSM [22]. 225 VCT clinics provide free HIV testing for MSM, FSWs, and other high-risk groups. Two HIV/AIDS designated hospitals to provide HARRT treatment for over 19,000 HIV infected people, and our VCT clinic is an outpatient department from one of the HIV/AIDS designated hospitals. The predominant HIV transmission route of annual newly reported HIV/AIDS cases in Shenyang was via the male-to-male sexual route, which accounted for 80.3% (712/887) cases in 2017. The reported HIV incidence was 6.9 (95% confidence interval (CI): 4.9–9.3)/100 person-years among MSM in Shenyang in 2013 [23, 24].

### Sample size and sampling procedure/technique

The MSM respondents were recruited via a mixed-method, including outreach recruitment by community volunteers in places such as bars, parks, and baths, peer referrals, and recruitment on gay websites and gay chat rooms. This study was conducted at the First Affiliated Hospital of China Medical University. The questionnaire was administered by professionally trained staff in one-to-one, face-to-face interviews. After obtaining written informed consent from each research participant, we collected their sociodemographic information, sexual behaviors, and RD behaviors from the questionnaire developed for this study (see Additional file 1 for the questionnaire). The sociodemographic information included age, place of residence, educational level, highest educational degree obtained, marital status, and ethnicity. Sexual behaviors included the main channel for finding sexual partners, sexual roles, and usage of condoms and nitrite inhalants during their most recent sexual intercourse. For RD behaviors in the most recent experience of anal intercourse, we asked MSM participants if they had cleansed their rectum before and/or after RAI, the type of liquid used for douching, the type of douching product, the reasons for RD, and their serosorting behavior, defined as the practice of agreeing to have unprotected anal intercourse only with partners of the same HIV status, which is becoming increasingly popular among MSM in general [7]. All MSM who participated in this study received a HIV consultation before and after HIV testing, as well as condoms, lubricant, and educational materials on HIV.

The required sample size was calculated using an estimation formula based on the difference between two sample rates for the cross-sectional survey study [25]. The significance level was 0.05, and power was 0.8. To compare the difference of HIV prevalence between the RD and non-RD group, the sample size calculated referred to a published study in which HIV prevalence was 4.3%(11/258) in the non-RD group and 14.9%(47/315) in RD group [11]. After calculations, each group should have a minimum sample of 121 participants. We utilized PASS (Power Analysis & Sample Size) software version 15 to calculate the sample size.

### Laboratory tests

Following completion of the questionnaire, 10 ml of venous blood was collected from each research participant for HIV-1 antibody screening, HIV-1 Pooled-RT-PCR, Western blot (WB), and *Treponema pallidum* (TP) testing.

HIV antibodies were screened using Biomérieux’s Human Immunodeficiency Virus (HIV 1/2) antibody diagnostic kit (ELISA method). Secondary screening was performed using Abbott Laboratories’ Human Immunodeficiency Virus Antibody Rapid Detection Kit (colloidal gold method). Once HIV-positive status was confirmed by the screening, the HIV confirmation test was performed using Gene Lab’s serum HIV Western blot (WB) method. Antibody-negative specimens were tested with the 24 mini-pool nucleic acid amplification test (NAAT). Blood samples giving a positive ELISA result but a negative or indeterminate WB result were tested with NAAT individually without mixing, using COBAS AMPLICOR HIV-1 MONITORTM Test, v1.5 diagnostic kit (Roche, 21,118,390,123), according to the COBAS AmpliPrep/COBAS TaqMan HIV-1 Test method [26].

For the detection of plasma TP, a rapid serotonin ring card test (RPR) was used for screening (Shanghai Kehua Bio-Engineering Co., Ltd.), and those who tested positive were further confirmed with *Treponema pallidum* particle agglutination (TPPA) (Fuji Corporation, Japan). Those who were screened and confirmed as positive were deemed to be TP-positive.

### Data analysis

Questionnaire and laboratory test results were recorded by two research assistants twice using EpiData 3.02 software until the data from both questionnaires were fully consistent. Descriptive statistical analysis was performed using mean ± standard deviation or the median and interquartile range (IQR) as a measure of statistical dispersion. Categorical variables were described using percentages of its frequency. Chi-square tests were used to compare differences in the categorical variables (e.g., marital status, education, and information related to the most recent anal sex). We used the median and interquartile range to describe the central tendency and dispersion of age, respectively.

Factors associated with HIV infection were analyzed using logistic regression models. We controlled for confounders in the multivariable logistic models and added one independent variable to the model each time. In the multivariable model, we controlled for age, education, and household registration. Independent variables with *p* < 0.2 in the univariate logistic model were entered into the multivariable model separately. We chose p < 0.2 as the cut-off point to include more RD-related behaviors that might be associated with HIV infection. The interaction between RD and CRAI was evaluated by the above multivariable logistic regression models. The inspection level α was set at 0.05. Statistical analysis was performed using SPSS 17.0 statistical software (IBM).

## Results

### Characteristics of the study participants

**Fig. 1:**
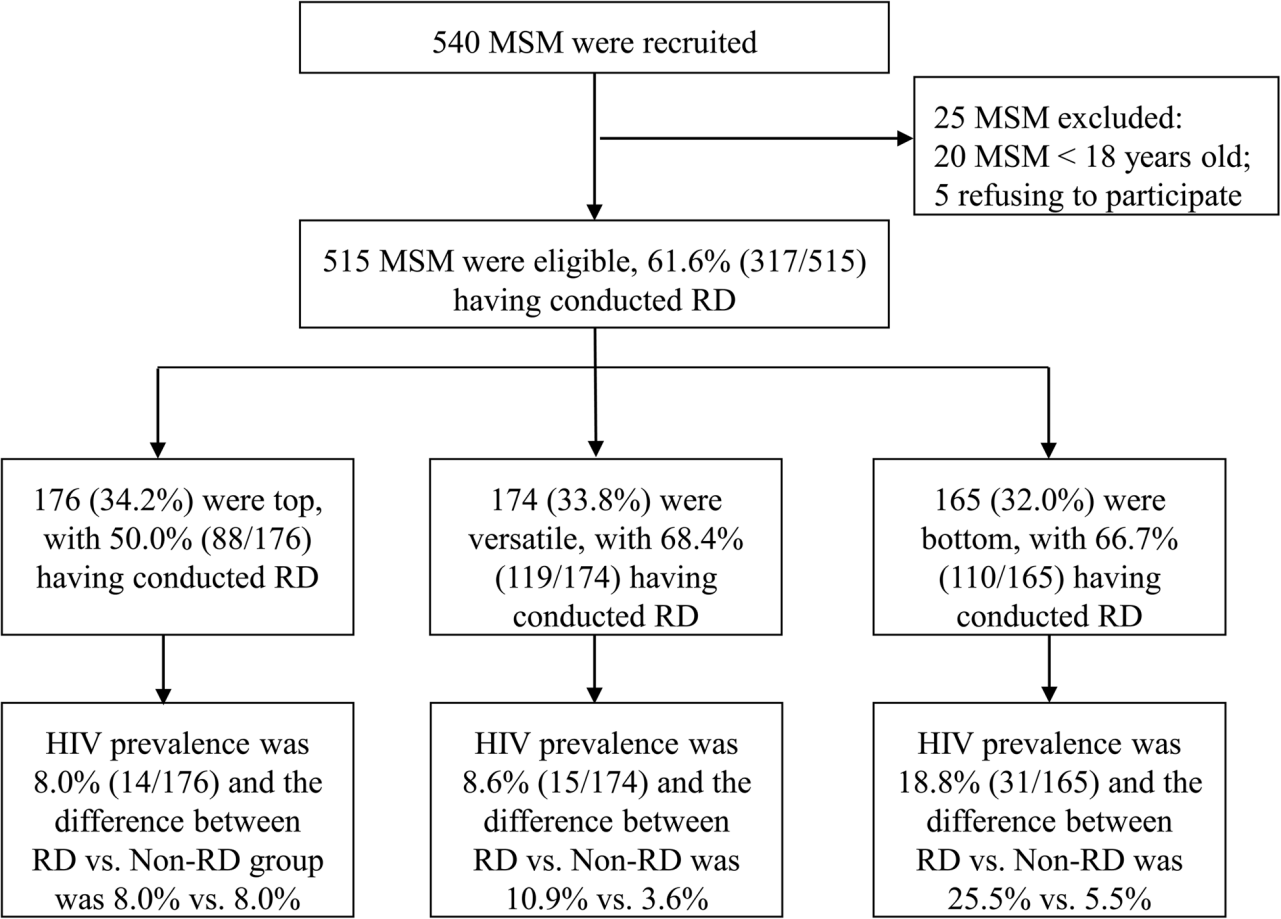
Flowchart of MSM study population, focusing on proportion of MSM using RD and HIV prevalence according to sexual role and use non-use of RD

In this study, 95.4% (515/540) of the MSM approached were eligible to participate in the cross-sectional study. The main reasons for exclusion were: (i) being underage (3.7%, 20/540) and (ii) 0.9% (5/540) refusing to participate in the research (Fig. 1). The median age among the research participants was 31 (IQR 25–37) years. If the time frame was the most recent anal sex, 28.3% (146/515) had CRAI, and 28.9% (149/515) consumed nitrite inhalants. Other demographic and sexual behaviors characteristics are given in Table 1.

**Table 1:** Characteristics of Shenyang MSM who either used or did not use rectal douching (RD) (*N* = 515)

Characteristics	Total (*n*, %)		Reported RD (*n*, %)		No reported RD (*n*, %)		*P*-value
Total	515	100.0	317	61.6	198	38.4	
Age (years)							
18–29	238	46.2	149	47.0	89	44.9	
≥ 30	277	53.8	168	53.0	109	55.1	0.649
Registered Liaoning Province residency							
No	88	17.1	47	14.8	41	20.7	
Yes	427	82.9	270	85.2	157	79.3	0.085
Education							
Junior high school and below	137	26.6	70	22.1	67	33.8	
Senior high school	114	22.1	69	21.8	45	22.7	
College and above	264	51.3	178	56.2	86	43.4	0.006
Marital status							
Single	307	59.6	184	58.0	123	62.1	
Married/divorced/cohabiting with female	101	19.6	63	19.9	38	19.2	0.593
Cohabiting with male partner	107	20.8	70	22.1	37	18.7	
Venue where sex is found							
Offline	197	38.3	111	35.0	86	43.4	
Online	318	61.7	206	65.0	112	56.6	0.056
Information related to most recent anal sex							
Main anal sexual role with males							
Versatile roles	174	33.8	119	37.5	55	27.8	
Bottom	165	32.0	110	34.7	55	27.8	
Top	176	34.2	88	27.8	88	44.4	0.001
CRAI							
Yes	146	28.3	111	34.1	35	17.7	
No	369	71.7	206	65.9	163	82.3	< 0.001
CIAI							
Yes	121	23.5	84	26.5	37	18.7	
No	394	76.5	233	73.5	161	81.3	0.042
Anal bleeding after anal sex							
No	491	95.3	296	93.4	195	98.5	
Yes	24	4.7	21	6.6	3	1.5	0.007
Used nitrite inhalants							
No	366	71.1	193	60.9	173	87.4	
Yes	149	28.9	124	39.1	25	12.6	< 0.001
Serosorting							
No	405	78.6	240	75.7	165	83.3	0.040
Yes	110	21.4	77	24.3	33	16.7	
Syphilis infection							
No	447	86.8	268	84.5	189	90.4	
Yes	68	13.2	49	15.5	19	9.6	0.056
HIV infection							
No	455	88.3	269	84.9	186	93.9	
Yes	60	11.7	48	15.1	12	6.1	0.002

### Rectal douching characteristics of the study participants

Among all of the research participants, 61.6% (317/515) reported douching their rectum at some time during their most recent anal intercourse, with 59.6% (307/515) conducting RD before sex and 38.4% (198/515) conducting RD after sex. Douching devices used before anal intercourse include a shower hose (85.3%, 262), commercial RD products (8.1%, 25), a bulb syringe (2.9%, 9), a syringe (1.6%, 5), a basin and water bottle (1.3%, 4), as well as other items (0.7%, 2). The types of douching liquids used (before/after RD, *n* = 317) included tap water (98.1%, 311), mixed solutions of water and soap (1.6%, 5), and commercial douching liquids (0.6%, 2). Almost all (97.4%, 299) of those who conducted RD reported doing so before anal sex for hygienic reasons, 23.1% (71) for feeling greater pleasure during sex, and 6.8% (21) for satisfying their sexual partner. The primary reason for RD post-anal intercourse was similar: 95.5% (189) did so in personal hygiene. Others douched their rectum after sex because their sexual partners did not use a condom during sex (14.1%, 28), and they felt that RD would prevent HIV infection (4.0%, 8).

Compared with RD non-users, RD users had: a higher level of education, were more likely to have had CRAI, were more likely to use nitrite inhalants, and were more likely to be living with HIV. Differences in the sociodemographic and behavioral characteristics among MSM who reported RD and those who did not are listed in Table 1.

### RD and other factors associated with HIV infection

Of the 515 MSM recruited, 60 men (11.7%) (95% CI: 9.0–14.7) tested HIV-1-positive and 68 men (13.2%) (95% CI: 10.4–16.4) tested syphilis-positive. None were detected HIV-1 screening negative but pooled NAAT positive. Univariate and multivariable logistic regressions were utilized to assess the factors associated with HIV infection (Table 2). The univariate logistic regression results indicated that HIV infection was statistically associated with RD during the most recent anal intercourse, RD before and after anal intercourse, playing the receptive sexual role (bottom), having condomless sex when playing the receptive role CRAI, being infected by syphilis, the interaction between RD and CRAI, and using nitrite inhalants before sex.

**Table 2:** RD and other factors associated with HIV infection among MSM using logistic regression model† (N = 515)

Characteristic	*N* = 515	HIV infection (%)	Crude OR (95% CI)	Model ^†^ AOR^#^ (95% CI)	*P*-value
Information related to most recent anal sex					
Main sexual role with males					
Top	176	14 (8.0)	1	1	
Bottom	165	31 (18.8)	2.7 (1.4–5.2)	2.4 (1.2–4.9)	0.010
Versatile	174	15 (8.6)	0.9 (0.5–2.3)	1.0 (0.5–2.3)	0.908
CRAI					
No	369	30 (8.1)	1.0	1.0	
Yes	146	30 (20.5)	2.9 (1.7–5.1)	2.7 (1.6–4.7)	< 0.001
CIAI					
No	394	41 (10.4)	1.0	1.0	
Yes	121	19 (15.7)	1.6 (0.9–2.9)	1.6 (0.9–2.9)	0.130
Serosorting					
Yes	110	8 (7.3)	1.0	1.0	
No	405	52 (12.8)	1.9 (0.9–4.1)	1.9 (0.9–4.2)	0.103
Rectal douching					
No	198	12 (6.1)	1.0	1.0	
Yes	317	48 (15.1)	2.8 (1.4–5.3)	2.8 (1.4–5.4)	0.003
RD × CRAI^&^					
Reference	404	33 (8.2)	1.0	1.0	
RD × CRAI	111	27 (24.3)	3.6 (2.1–6.3)	3.5 (2.0–6.2)	< 0.001
Rectal douching before sex					
No	208	15 (7.2)	1.0	1.0	
Yes	307	45 (14.7)	2.2 (1.2–4.1)	2.2 (1.2–4.2)	0.012
Rectal douching after sex					
No	317	29 (9.1)	1.0	1.0	
Yes	198	31 (15.7)	1.8 (1.1–3.2)	1.9 (1.1–3.2)	0.026
Equipment used for RD					
Commercial tools	48	3 (6.3)	1.0	1.0	
Shower hose	270	45 (16.7)	3.0 (0.9–10.1)	3.3 (1.0–11.3)	0.053
No RD	197	12 (6.1)	1.0 (0.3–3.6)	1.1 (0.3–3.9)	0.941
Douching liquids					
Top water	311	46 (14.8)	1.0	1.0	
Water + soap	5	2 (40.0)	3.8 (0.6–23.6)	5.8 (0.8–41.2)	0.076
Vaginal/traditional Chinese medicine lotion	2	0 (0.0)	NA	NA	0.999
No RD	197	12 (6.1)	0.4 (0.2–0.7)	0.4 (0.2–0.7)	0.004
Used nitrite inhalants					
No	364	32 (8.8)	1.0	1.0	
Yes	149	28 (18.8)	2.4 (1.4–4.2)	2.6 (1.5–4.5)	0.001
Syphilis infection					
No	447	47 (10.5)	1.0	1.0	
Yes	68	13 (19.4)	2.0 (1.0–4.0)	2.1 (1.1–4.2)	0.033

†: Model was constructed by controlling the fixed covariates (age, education, and household registration). Covariates that were significant at *p* < 0.20 in the univariable model were then considered in a multivariable model

#: AOR, adjusted odds ratio

&: the interaction effect between RD and CRAI was shown in the table

**Fig. 2:**
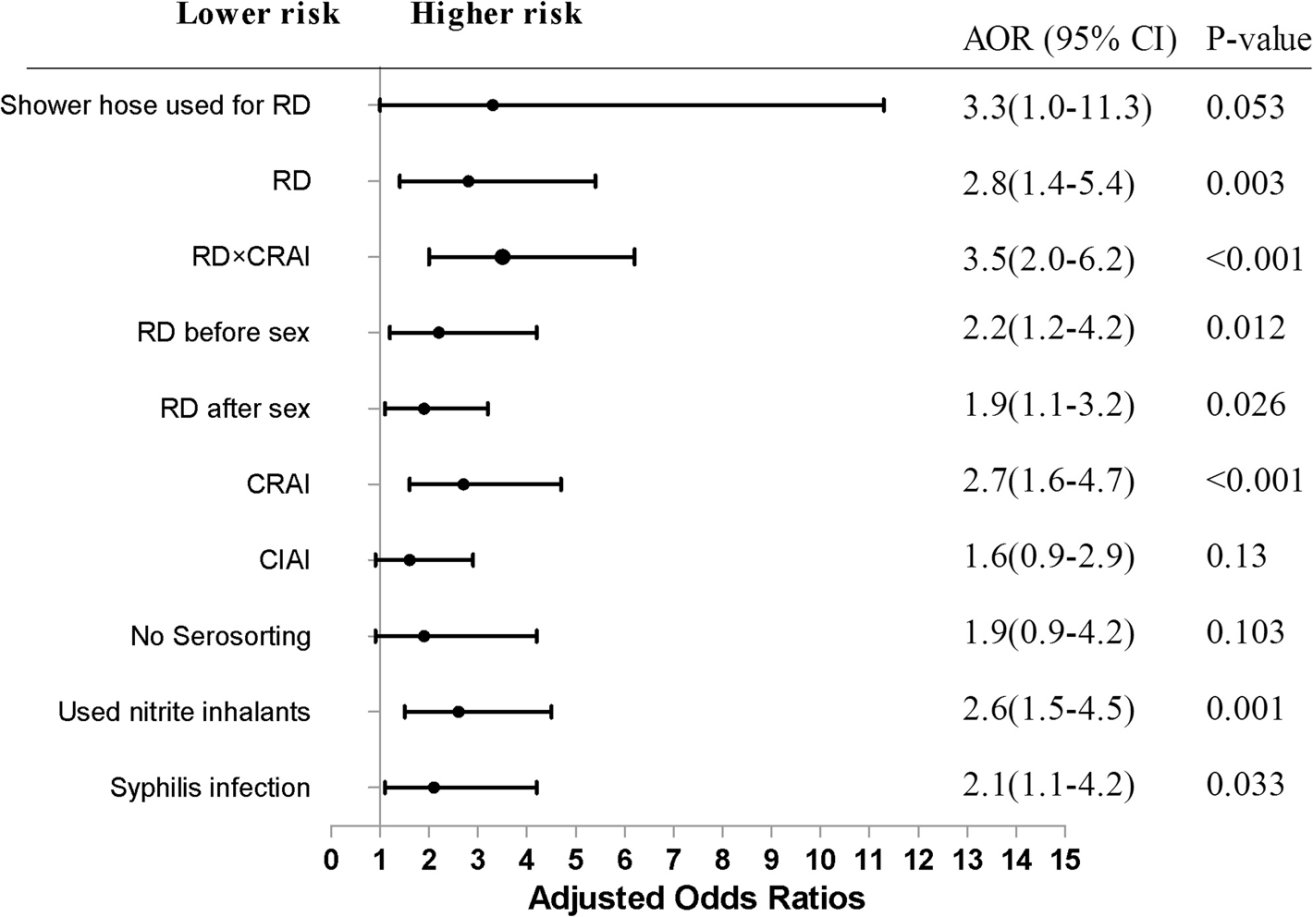
AORs and 95% CI between high-risk behavior and HIV infection among MSM participants HIV infection-related factors were analyzed using multivariable logistic regression model analysis. Multivariable logistic Model controlled for fixed covariates (age, education, and household registration). Abbreviations RD, rectal douching; CRAI, condomless receptive anal intercourse; CIAI, condomless insertive anal intercourse.

According to Table 2, in multivariable logistic regression analysis revealed that variables that were statistically associated with HIV infection in the most recent anal intercourse included: RD (adjusted odds ratio (AOR), 2.8; 95% CI, 1.4–5.4), practicing RD before sex (AOR, 2.2; 95% CI, 1.2–4.2), the interaction between RD and CRAI (AOR = 3.5; 95% CI, 2.0–6.2), using a shower hose for RD (AOR, 3.3; 95% CI, 1.0–11.3) and other risk factors, practicing RD after sex, CRAI, using nitrite inhalants before anal sex in the most recent anal sex main sexual role with males as bottom and syphilis infection (Fig. 2).

## Discussion

This cross-sectional study of 515 MSM in Shenyang, China, reports the proportion of and reasons for practicing RD among MSM in China. It investigated the impacts of RD products, douching liquid types, and the timing of RD on HIV infection. We found that using a shower hose (inappropriate douching tools) to assist RD was positively associated with the odds of HIV-1 infection among MSM. Meanwhile, we found the interaction between RD and CRAI was associated with HIV infection. This finding contributes to our understanding of RD behavior and the risks of HIV infection among MSM. It also helps to clarify the possible reasons for the high HIV disease burden of MSM in China, and our study provides first-hand evidence to include in future publicity materials and educational activities about the risks associated with RD and HIV infection among MSM.

This study found that the proportion of RD among MSM in Shenyang, a city with a lower level of GDP than other cities in China, was over 60%, similar to the percentage reported for Beijing (59.3%) [27]. These figures suggest that RD might be a common practice among MSM in urban China whatever their economic level. The prevalence of RD behavior among MSM in China is close to that of the USA (66%), Kenya (63%) [12, 13], the UK, Brazil, and France (53.4–54.3%) [11, 14, 15], but higher than that in Peru (18.2–27.0%) [28, 29] and the Netherlands (13.6–46%) [30, 31]. Despite this high prevalence among MSM worldwide, scientific data for and knowledge of RD in the Chinese MSM population is still insufficient. Around a quarter of MSM in the USA did not know how to douche the rectum correctly [16], and more than 94% of MSM in Brazil had not received any professional instruction [14]. Currently, there is no authoritative information on RD for MSM in China. The disparity between the prevalence of RD and the lack of instruction about it highlights the importance of delivering accurate and relevant information about RD to the MSM population via social media.

This study found that RD is positively correlated with the odds of HIV infection (AOR = 2.8) in MSM participants, consistent with previous findings from the USA and Europe and a recent meta-analysis [16, 17, 21, 31, 32]. We found inappropriate tools used for rectal douching is associated with higher HIV infection risk in MSM participants, compared with the commercial equipment. The most common inappropriate tool used for RD in MSM participants was shower hose. We also found that the use of a shower hose associated with increased odds of HIV infection. Shower hoses are not expressly designed for RD as they have irregular edges. The process of using a shower hose to complete RD may cause damage to the perianal skin, and hence increase the risk of HIV infection. More than 85% of MSM participants used the shower hose to flush their anus in this survey, which is significantly higher than in foreign MSM populations. According to our study results, health workers and MSM community based organizations (CBOs) should widely publicize the potential for this type of RD to increase HIV infection in the MSM community. Besides, this study found that most MSM conducted pre-anal intercourse RD using tap water. Due to the difference in fluid osmotic pressure inside and outside the rectal epithelial cells, the latter are in a fully filled state following RD, and the risk of rupturing them is increased during anal sex. Water-based enema solutions are typically hypotonic and exert a lower osmotic pressure compared to the contents of the colon. As a result, excess water may be absorbed by epithelial cells, leading to water toxicity and cell lysis [20]. So enemas consisting of saline solution are recommended for MSM who practice RD frequently. For the benefit of MSM who engage in RD, it is recommended that they receive proper educational materials on how to select the most appropriate douching products and liquids so as to reduce the risk of HIV and sexually transmitted infections (STIs) resulting from rectal mucosal injuries.

This study found that, compared to MSM who do not perform RD, MSM who do use RD engaged in a greater proportion of bottom and versatile roles during anal sex.. The bottom and versatile sexual roles has different risk of HIV infection compared with the top role [33], hence controlling the sexual role may help well explain the association between RD and HIV infection. The results of our analysis have given us more reliable evidence of the association between RD behavior and HIV infection in the MSM population.

Understanding the reasons why MSM practice RD is important for promoting HIV-related education and conducting further studies. Over 95% of MSM participants did so because they wanted to remain clean and hygienic before and/or after anal intercourse and increase pleasure during sex. A proportion (14.6%) were under the misconception that RD could wash semen away from the rectum and prevent HIV infection and STIs. This suggests that MSM participants were very concerned about their health and preventing HIV infection and other sexually transmitted diseases (STDs). Given the positive correlation between RD behavior and a willingness to use rectal microbicides among Peruvian MSM [29], our study findings indicate that Chinese MSM are likely to be good candidates to be recruited to use rectal microbicide enemas for HIV prevention.

This study also found that factors contributing to HIV-1 infection include: (i) condomless anal intercourse; (ii) using nitrite inhalants before anal intercourse; and (iii) not serosorting in the most recent anal intercourse and main sexual role with males as bottom. These findings are consistent with those of international peer reports [17, 34]. It is worth noting that previously there was a lack of evidence to support the implementation of serosorting behavior to prevent HIV infection in MSM in China, although the Chinese Center for Disease Control and Prevention (China CDC) had issued related serosorting guidelines for MSM in 2016. Hence, in China, pertinent interventions, such as promoting serosorting among MSM who practice RD and partner notification promotion, are urgently needed. The results of this study will help China and other countries in a similar situation to promote serosorting measures among MSM to reduce HIV infection.

### Strengths of the study

This study demonstrates an association between RD products’ types and HIV infection among MSM, providing empirical evidence for informing future sexual health education efforts. Further, our study demonstrated that RD and CRAI interact with each other in promoting HIV infection, and MSM with both higher RD practice and CRAI behavior were more likely to have HIV infection. Previous studies have also found RD associated with CRAI [11, 13]. It is recommended that condom use promotion is the most critical behavioral interventions among MSM practice RD before or after sex.

### Limitations of the study

In order to reduce the effects of recall bias, this study only collected information on RD behavior during the most recent sexual experience. It did not analyze the cumulative effect of RD frequency and timing on HIV infection. Additionally, sampling technique used in our study may affect the representativeness of the sample, MSM came to our VCT clinic might have higher health awareness and research participants from the sample pool all came from 1 of 225 VCT clinics in Shenyang, and thus were not representative of other areas in Shenyang and China. Third, the questionnaire has not been tested for reliability and validity before, so interpreting related study results should be cautious. Lastly, the cross-sectional study design used in this study cannot determine the cause-effect relationship between RD and the risk of HIV infection, for which further prospective cohort studies should be undertaken.

## Conclusions

RD behavior is prevalent among MSM in Shenyang in northeastern China. Because RD, CRAI, the interaction effect of RD and CRAI, might increase the odds of HIV infection, it is suggested that relevant health information targeting MSM who have used shower hoses for RD before/after sex and who have CRAI should be promoted. This may help MSM to understand the association between RD and HIV infection to reduce the spread of HIV among the MSM population. Moreover, safer types of douching products and liquids should be formulated in order to contain a potential new channel of HIV dissemination.

## Supplementary Information

**Additional file 1.** The questionnaire contains sociodemographic information, sexual behaviors, and RD behaviors information.

## Data Availability

The data bank analyzed during this study are available from the corresponding authors on reasonable request.

## Abbreviations

CAI: Condomless anal intercourse
CDC: (China) Center for Disease Control and Prevention
CIAI: Condomless insertive anal intercourse
CRAI: Condomless receptive anal intercourse
ELISA: Enzyme-linked immunosorbent assay
GDP: Gross domestic product
HIV: Human immunodeficiency virus
IQR: Interquartile range
IRB: Institutional review board
MSM: Men who have sex with men
NAAT: Nucleic acid amplification testing
RAI: Receptive anal intercourse
RD: Rectal douching
RPR: Rapid serotonin ring card test
TP: Treponema pallidum
TPPA: Treponema pallidum particle agglutination
WB: Western blot
