# Supplementary material for: The use of inappropriate anal douching tool associates with increased HIV infection among men who have sex with men: a cross-sectional study in Shenyang, China
Source: BMC Public Health. 2021 Jan 28;21:235. doi: 10.1186/s12889-021-10276-z (PMC7844917; doi:10.1186/s12889-021-10276-z)
Supplement: Supplementary file 1 — Additional file 1. The questionnaire contains sociodemographic information, sexual behaviors, and RD behaviors information. [file 12889_2021_10276_MOESM1_ESM.docx]

**Questionnaire (English)**

**Survey on rectal douching among men who have sex with men in Shenyang, China**

| **PID.**：**________________**  **Part 1 Socio-demographic characteristics.** |
| --- |
| - 1. Your age: ________(years) |
| 1-2 Province of permanent residence (Hukou):   1. Liaoning province (2) Other provinces (_________) |
| 1-3 The highest education attained:   1. Primary school or lower (2) Junior high school (3) Senior high school (or equal academic qualification) (4) College (5) University or above |
| 1-4 Marital status:  (1) Single (2) Cohabiting with male partner (3) Cohabiting with woman (4) Married with woman (5) Divorced (6) Widowed (7) Others: ______________ |
| 1-5 What is your nationality?  (1)The Han nationality (2) Others: ______________ |

| **Part 2：Sex behavior during most recent anal sex.** |
| --- |
| 2-1 How did you find your last sexual partner(s)?  (1) Online (QQ, WeChat, Blued, Other dating software)  (2) Offline (park, bathroom, bar or Other places) |
| 2-2 What was your sexual role with your last male partner?  (1) Top (2) Bottom (3) Versatile roles |
| 2-3 Did you use a condom during last anal sex as bottom role?   1. Yes (2) No (3) No such behavior |
| 2-4 Did you use a condom during last anal sex as top role?   1. Yes (2) No (3) No such behavior |
| 2-5 Did you find yourself any anal bleeding after last anal sex?   1. Yes (2) No (3) No such behavior |
| 2-6 Did you use Rush poppers (nitrite inhalants) during last anal sex?   1. Yes (2) No (3) No such behavior |
| 2-7 What was the HIV status of your last male partner?   1. Negative (2) Positive (3) I don’t know |
| **Part 3：Rectal douching information related to most recent anal sex.** |
| 3-1 Did you insert cleaning fluids into your anus before last anal sex?   1. Yes (2) No (3) No such behavior |
| 3-2 Why did you insert cleaning fluids into your anus before last anal sex? [you can choose more than one answer]   1. Cleaning/hygiene (2) More pleasurable anal intercourse (3) Satisfying their sexual partner (4) Constipation (5) Others |
| 3-3 Why did you insert cleaning fluids into your anus after last anal sex? [you can choose more than one answer]   1. Cleaning/hygiene (2) More pleasurable anal intercourse (3) Satisfying their sexual partner (4) Constipation (5) Others |
| 3-4 What equipment did you use to assist fluids into your anus (enema/ Rectal douching)?  (1) Shower hose (2) Commercial tools (3) Others (4) No such behavior |
| 3-5 What douching liquids did you use for enema/ Rectal douching?  (1) Tap water (2) homemade solutions (water + soap) (3) Vaginal/traditional Chinese medicine lotion (4) Others (5) No such behavior |

**(End of the survey, thanks for your support!)**
